# The Impact of an Intervention to Improve Caregiver Contribution to Heart Failure Self-care on Caregiver Anxiety, Depression, Quality of Life, and Sleep

**DOI:** 10.1097/JCN.0000000000000998

**Published:** 2023-05-18

**Authors:** Giulia Locatelli, Paola Rebora, Giuseppe Occhino, Davide Ausili, Barbara Riegel, Andrea Cammarano, Izabella Uchmanowicz, Rosaria Alvaro, Ercole Vellone, Valentina Zeffiro

**Affiliations:** **Giulia Locatelli, MSN, RN** PhD Student, Department of Biomedicine and Prevention, University of Rome Tor Vergata, Italy; and PhD Student, School of Nursing, Midwifery and Paramedicine, Faculty of Health Sciences, Australian Catholic University, Melbourne, Australia.; **Paola Rebora, PhD, MS** Associate Professor, School of Medicine and Surgery, University of Milano-Bicocca, Monza, Italy.; **Giuseppe Occhino, MS** PhD Student, School of Medicine and Surgery, University of Milano-Bicocca, Monza, Italy.; **Davide Ausili, PhD, RN** Associate Professor, Department of Medicine and Surgery, University of Milano-Bicocca, Monza, Italy.; **Barbara Riegel, PhD, RN, FAAN, FAHA** Professor, University of Pennsylvania School of Nursing, Philadelphia; Professorial Fellow, Mary MacKillop Institute for Health Research, Australian Catholic University, Melbourne, Australia; and Co-Director, International Center for Self-Care Research.; **Andrea Cammarano, MS** Adjunt Professor, Department of Biomedicine and Prevention, University of Rome Tor Vergata, Italy; **Izabella Uchmanowicz, PhD, RN, FESC, FHFA** Professor, Department of Nursing and Obstetrics, Faculty of Health Sciences, Wroclaw Medical University; and Institute of Heart Diseases, University Hospital, Wroclaw, Poland; **Rosaria Alvaro, MSN, RN, FESC, FAAN** Professor, Department of Biomedicine and Prevention, University of Rome Tor Vergata, Italy.; **Ercole Vellone, PhD, RN, FESC, FAAN** Associate Professor, Department of Biomedicine and Prevention, University of Rome Tor Vergata, Italy; and Visiting Professor, Wroclaw Medical University, Poland.; **Valentina Zeffiro, PhD, RN** Assistant Professor, Department of Biomedicine and Prevention, University of Rome Tor Vergata, Italy.

**Keywords:** anxiety, caregivers, depression, heart failure, motivational interviewing, quality of life, sleep

## Abstract

Better caregiver contribution to self-care in heart failure is associated with better patient outcomes. However, caregiver contribution to self-care is also associated with high anxiety and depression, poor quality of life, and poor sleep in caregivers. It is still unclear whether interventions that encourage caregivers to contribute more to patient self-care might increase caregivers' anxiety and depression and decrease their quality of life and sleep.

**Objective:**

The aim of this study was to assess the impact of a motivational interview intervention aimed at improving caregiver contribution to self-care in heart failure on caregivers' anxiety, depression, quality of life, and sleep.

**Methods:**

This is a secondary outcome analysis of the MOTIVATE-HF trial. Patients with heart failure and their caregivers were randomized into arm 1 (motivational interview to patients), arm 2 (motivational interview to patients and caregivers), and arm 3 (standard care). Data were collected between June 2014 and October 2018. The article has been prepared following the Consolidated Standards of Reporting Trials checklist.

**Results:**

A sample of 510 patient-caregiver dyads was enrolled. Over the year of the study, the levels of anxiety, depression, quality of life, and sleep in caregivers did not significantly change among the 3 arms.

**Conclusions:**

Motivational interview aimed at improving caregiver contribution to self-care does not seem to increase caregiver anxiety and depression, nor decrease their quality of life and sleep. Thus, such an intervention might be safely delivered to caregivers of patients with heart failure, although further studies are needed to confirm our findings.

Despite improvements in prevention, diagnosis, and treatments, outcomes such as physical and psychological symptoms, quality of life (QoL),^1^ use of emergency services, hospitalization, and mortality rates^2^ remain poor in patients with heart failure (HF).^3^ These outcomes may improve if patients perform HF self-care,^4,5^ which has been defined as a set of behaviors to maintain physiological and emotional stability (self-care maintenance), detect and interpret symptoms (symptom perception), and respond to symptoms when they occur (self-care management).^6^ However, patients find it challenging to perform self-care.^7^ In these cases, caregiver contribution (CC) to patient self-care may be crucial to improve both patient self-care and patient outcomes.^8^

## Background

Caregiver contribution to HF self-care has been defined as the process of recommending behaviors to patients to (1) maintain HF stability (CC to self-care maintenance), (2) monitor HF symptoms (CC to symptom monitoring), and (3) respond to the HF-related signs and symptoms when they occur (CC to self-care management).^9^ Higher CC to HF self-care has been found to be associated with better patient outcomes, such as better adherence to fluid restriction and medications, exercise, diet, and flu vaccination,^10^ and lower mortality rates and rehospitalizations.^11^ At the same time, CC has also been found to be associated with higher anxiety, worsening depression, poor QoL,^12^ and poorer sleep^13^ in caregivers themselves. Consequently, interventions that encourage caregivers to further contribute to patient self-care might increase caregivers' anxiety and depression and decrease their QoL and sleep.

To our knowledge, only 1 previous trial has evaluated the impact of an intervention aimed at improving CC to HF self-care on caregiver anxiety, depression, and QoL, and none has evaluated the impact of similar interventions on caregivers' sleep. Wingham and colleagues^14^ explored whether a 12-week rehabilitation intervention improved CC to self-care and caregiver self-efficacy while also assessing the impact of such an intervention on caregiver anxiety, depression, and QoL. At 12 months from enrollment, the authors observed that caregiver self-efficacy significantly increased in the intervention group, but the intervention did not affect CC to self-care, anxiety, depression, or QoL. If these results were confirmed by other studies, it would strengthen the probability that interventions aiming to improve CC to self-care, besides improving patient outcomes, do not worsen caregiver outcomes.

In the MOTIVATE-HF trial,^15^ we recruited patients with HF and their caregivers and randomized them into 3 arms. Some of the caregivers received a motivational interviewing (MI) intervention, whereas some others did not. Therefore, in this secondary outcome analysis of the MOTIVATE-HF randomized controlled trial (RCT), we compared changes in caregivers' anxiety, depression, QoL, and sleep among the 3 study arms to explore whether receiving the MI intervention (aiming to increase CC to self-care) had an influence on these variables.

## Methods

This study is a planned secondary outcome analysis of the MOTIVATE-HF RCT,^15^ which focused on improving self-care in patients with HF using MI. That RCT randomized participants into 3 arms: arm 1, MI for patients only; arm 2, MI for patients and their caregivers; and arm 3, standard care. The study protocol^16^ was previously registered on Clinicaltrials.gov (identifier: NCT02894502). In this planned secondary outcome analysis, we compared changes in caregivers' anxiety, depression, QoL, and sleep among the 3 study arms to explore whether receiving the MI intervention to increase CC to self-care influenced these variables or not.

The intervention, delivered by specially trained nurses (ie, 40-hour training course on MI and HF evidence-based care), consisted of a 60-minute face-to-face MI session followed by 3 telephone contacts within 2 months from enrollment.^15^ During the MI session, the interventionists, guided by the principles of MI,^17,18^ performed the intervention with the patients (arm 1) or both the patients and caregivers (arm 2). In particular, the interventionists (1) developed the discrepancy between the current behaviors adopted by patients and caregivers, and the evidence-based behaviors that are needed to maintain HF under control; (2) expressed empathy and support; (3) stimulated participants' problem solving and self-efficacy; and (4) avoided arguing with participants regarding their choices. This approach was used only for patients in arm 1 and both for patients and caregivers in arm 2. Within 2 months from the face-to-face MI intervention, the interventionists had 3 telephone contacts with the participants to booster the initial intervention. The telephone contacts were guided by the MI principles too, and they included patients only (arm 1) or patients and caregivers (arm 2). All study arms, including the standard care, received educational materials on HF self-care.

Patients and caregivers were randomized to one of the 3 study arms by a research assistant blinded to the other step of the trial. A block randomization scheme of 15 patient-caregiver dyads was generated and followed a 1:1:1 ratio in the 3 study arms. Research assistants collecting data were blinded to the study arm assignment. More details on the randomization procedures can be found in the original article.^15^

This article and its findings have been reported following the Consolidated Standards of Reporting Trials guidelines.

### Participants

In the MOTIVATE-HF trial,^15^ patients with HF and their caregivers were recruited from 3 healthcare centers in Italy (an hospital ward, an outpatient clinic, and a community setting). Inclusion criteria for patients were (1) an HF diagnosis with a New York Heart Association class between II and IV, (2) poor self-care with a score between 0 and 2 on at least 2 items of the Self-Care Maintenance or Self-Care Management scale of the Self-Care of Heart Failure Index v.6.2, and (3) willingness to participate in the study and sign the consent form. Patients were excluded if they (1) had a severe cognitive impairment with a score between 0 and 4 on the Six-item Screener, (2) had a myocardial infarction in the previous 3 months, or (3) lived in a residential facility where self-care was an unreasonable expectation. Caregivers were enrolled when patients identified them as the persons providing most of their informal care. At enrollment, if a member of the patient-caregiver dyads did not want to participate in the study, they were both excluded. At follow-ups, if a member of the dyads refused to continue in the study, the other member was kept enrolled if possible.

### Data Collection

Data were collected between June 2014 and October 2018. After the study protocol^16^ received ethical approval, participants were approached by a research nurse explaining the study aims. If both patient and caregiver were willing to participate to the study and to sign the informed consent form, the research assistant screened the patient with the Self-Care of Heart Failure Index v.6.2 and the Six-Item Screener. If meeting the inclusion criteria, both patients and caregivers were given baseline questionnaires to complete individually. All instruments were administered at baseline (T0) and at 3 (T1), 6 (T2), 9 (T3), and 12 (T4) months from enrollment by research assistants blinded to the study arms. At baseline, data were collected at the study centers, whereas follow-up data were collected telephonically by research assistants who were blinded to the study arms. Participants were not blinded to the study arms.

A battery of psychometrically sound instruments was used in the trial, but we only considered the following ones for this analysis. Caregivers' anxiety and depression were measured using the Hospital Anxiety and Depression Scale (HADS).^19^ The HADS consists of 2 scales, 1 for anxiety (the Hospital Anxiety Scale [HAS]) and 1 for depression (the Hospital Depression Scale [HDS]), with 7 items each. Scores of both scales range between 0 and 21, with higher scores indicating higher anxiety and depression. This instrument has shown supporting validity and reliability in caregivers.^20^ In this study, reliability of the HADS at baseline resulted with a Cronbach *α* of 0.83 for both the HAS and HDS.

Quality of life of caregivers was measured using the 12-item Short Form Health Survey (SF-12),^21^ a self-report instrument. It includes 2 domains: the physical component summary (PCS) and the mental component summary (MCS). Scores of the 2 dimensions range between 0 and 100, with higher scores indicating better QoL. The SF-12 has been recently found to be valid and reliable.^22^ In this study, the reliability of the PCS and MCS resulted in Cronbach *α*s of 0.79 and 0.70, respectively.

Caregivers' sleep quality was measured using the Pittsburgh Sleep Quality Index (PSQI),^23^ a self-reported questionnaire assessing sleep quality and disturbances over the last month. The PSQI is composed of 19 items, assessed on a Likert scale ranging from 0 (“not during the past month”) to 3 (“3 or more times a week”), which are combined to form 7 “component” scores. The sum of all scores yields 1 global score ranging from 0 (no difficulty) to 21 (severe difficulties). A recent study showed that the PSQI has good psychometric characteristics also in informal caregivers.^24^ In this study, Cronbach *α* was 0.78.

### Ethical Considerations

This study received ethical authorization from the university ethical board.

### Data Analysis

The power calculation estimated a sample size of 480 patients for the MOTIVATE-HF RCT based on self-care maintenance, the primary end point, while accounting for an estimated 50% attrition rate. The actual sample size of each arm for this study was estimated to achieve a power of at least 75% to detect an effect size of 0.3, with a significance level of .05 using a 2-sided 2-sample *t* test. Considering a common standard deviation (SD) of 5, an effect size of 0.3 would result in a minimal difference of 1.5 that is compatible with HADS and PSQI end points.^25,26^ As far as SF-12, if we considered a clinically significant change of 5^27^ with an SD of 10 (effect size, 0.5), we would reach a power of 99%.

Caregivers' baseline characteristics were summarized by arm as medians and quartiles (Q1–Q3) or as means and SDs for continuous data, and as absolute numbers and frequencies (%) for categorical data. Changes in anxiety and depression (HADS), physical and mental QoL (SF-12), and sleep quality (PSQI) scores during follow-up were reported as the difference (Δ) of these scores at each follow-up time (T1, T2, T3, and T4) minus the baseline scores (T0). Two-sample *t* tests were used to compare the difference in scores in arm 2 with respect to both arms 1 and 3. Changes over time (from T0 to T4) in the scores were analyzed with mixed models to account for dropout and missing values. As response variables, we included the HADS, SF-12, and PSQI scores available from T0 to T4 for each caregiver. The dependence between HADS, SF-12, and PSQI scores on the same subject was accounted for by including a random intercept and slope in each model. The models included the follow-up visit (as a continuous variable), the randomization arm (as a categorical variable, with arm 2 as reference), the interaction between the randomization arm and the follow-up visit, and the “living with the patient” condition as covariates.

### Treatment Fidelity

Treatment fidelity in the intervention arms (1 and 2) was assessed through 2 steps. In the first step, the technical and relational components of MI were evaluated using the Motivational Interviewing Integrity Scale,^18^ which ranges from 1 (lowest MI quality) to 5 (highest MI quality). Ideally, adequate quality scores are those ≥3 and ≥4 for the technical and relational dimensions, respectively. We randomly selected and assessed 48 audiotapes from arm 1 and 97 audiotapes from arm 2, obtaining a mean (SD) score of 2.4 (0.5) and 2.8 (0.8) for the technical and relational dimensions, respectively. In the second step, we assessed whether all the follow-up telephone calls were conducted as planned,^16^ and they were.

## Results

### Caregivers' Characteristics, Hospital Anxiety and Depression Scale, 12-Item Short Form Health Survey, and Pittsburgh Sleep Quality Index at Baseline

The complete sociodemographic characteristics of patients and caregivers enrolled in the MOTIVATE-HF and the participant flow are published in the original article.^15^ Briefly, we screened 1032 dyads of patients with HF and caregivers for eligibility and randomized 510 dyads in the 3 study arms. At baseline, caregivers in the 3 arms had comparable characteristics, except for the variables “caregiver living with the patient” (*P* = .001) and “relationship with the patient” (*P* = .021). In arm 2, a higher percentage of caregivers were living with the patients, mainly being their spouses. In arm 1, most caregivers were the children of the patients, whereas in arm 3, caregivers were equally spouses or children of the patients. Their median age was 55 years; they were mainly female (76%), married (72%), educated at a high level (55%), employed (73.5%), and living with the patient (60%) (Table 1). Among the 3 arms of the study, a portion between the 72.2% and 81.9% of the caregivers were patients' spouses and children. These caregivers cared for patients with HF with a median age of 74 years, who were mainly male (57.7%), retired (76.2%), and in New York Heart Association class II (61.9%).^28^

**TABLE 1:** Caregivers' Characteristics at Baseline (N = 510)

Characteristics	Missing	Arm 1: MI Only for Patients (n = 155)	Arm 2: MI for Patients and Caregivers (n = 177)	Arm 3: Usual Care for Patients and Caregivers (n = 178)
Age, median (IQR), y	9	54 (44–64)	57 (44–68)	53 (42–64)
Sex (male), n (%)	7	36 (24.0)	42 (23.9)	45 (25.4)
Marital status, n (%)	8			
Married		108 (72.5)	124 (70.5)	129 (72.9)
Widower		6 (4.0)	3 (1.7)	3 (1.7)
Divorced		10 (6.7)	14 (8.0)	12 (6.8)
Single		25 (16.8)	35 (19.9)	33 (18.6)
Education (high school or higher), n (%)	9	90 (56.9)	86 (49.4)	99 (56.3)
Employment (retired), n (%)	7	33 (22.0)	50 (28.4)	52 (29.4)
Caregiver living with patient,^a^ n (%)	8	76 (51.0)	126 (71.6)	104 (58.8)
Relationship with the patient^b^	7			
Child		70 (46.7)	62 (35.2)	64 (36.2)
Spouse		43 (28.7)	82 (46.6)	64 (36.2)
Brother/sister		5 (3.3)	4 (2.3)	8 (4.5)
Friend		7 (4.7)	3 (1.7)	3 (1.7)
Other		25 (16.7)	25 (14.2)	38 (21.5)
Hospital Anxiety Scale, mean (SD)	7	7.7 (4.5)	7.3 (4.4)	7.5 (4.7)
Hospital Depression Scale, mean (SD)	7	5.7 (4.5)	5.9 (4.1)	6.1 (4.6)
SF-12 physical component summary, mean (SD)	7	49.2 (8.0)	48.8 (8.5)	48.3 (8.3)
SF-12 mental component summary, mean (SD)	7	48.1 (9.1)	49.6 (9.4)	48.0 (9.1)
Global PSQI score, mean (SD)	16	9.8 (3.6)	10.1 (3.5)	9.7 (3.0)

Abbreviations: IQR, interquartile range; MI, motivational interviewing; PSQI, Pittsburgh Sleep Quality Index; SF-12, 12-item Short Form Health Survey.

^a^Test on the difference among the 3 arms, *P* = .001.

**^b^**Test on the difference among the 3 arms, *P* = .021.

Among the 3 arms, HAS mean scores were low, ranging from 7.3 and 7.7^29^; HDS mean scores also were low, ranging between 5.7 and 6.1.^29^ Furthermore, SF-12 PCS mean scores ranged between 48.3 and 49.2, and SF-12 MCS mean scores ranged between 48.0 and 49.6, slightly lower than the normative mean value (50.0),^30^ indicating average physical and mental QoL. Finally, PSQI mean scores ranged between 9.7 and 10.1, indicating poor sleep quality.^23^ All these scores were comparable among the 3 study arms.

### Caregiver Anxiety and Depression at Follow-ups

The changes (Δ) in caregiver anxiety and depression at each follow-up in each study arm are shown in Table 2. Over the 12 months of the study, anxiety and depression decreased over time, but without significant differences among the 3 arms. Model-based trends showed that, over the year of the study, caregiver anxiety and depression scores decreased by approximately half-point for each visit (decrease of HAS scores in arm 2: 0.45 [95% confidence interval (CI), −0.67 to −0.24], *P* < .0001; decrease of HDS scores in arm 2: 0.39 [95% CI, −0.60 to −0.18], *P* = .0003), but the decrement was not different among the 3 arms (Table 3). In caregivers involved in MI (arm 2), the difference with respect to arm 1 (MI only for patients) was 0.04 (95% CI, −0.28 to 0.36; *P* = .8160) in HAS and 0.05 (95% CI, −0.27 to 0.36; *P* = .7659) in HDS. Over the year of the study, caregivers living with the patient had higher HAS scores, although the difference was not statistically significant (0.63; 95% CI, −0.05 to 1.30; *P* = .07), whereas they had a significant increase in HDS of nearly 1 point (0.81; 95% CI, 0.17–1.45; *P* = .01; Table 3) compared with caregivers not living with the patient.

**TABLE 2:** Caregivers' Hospital Anxiety and Depression Scale, Physical and Mental 12-Item Short Form Health Survey Scores, and Global Pittsburgh Sleep Quality Index Score Changes During Follow-ups by Difference With the Values at T0

Variable	N	Arm 1: MI Only for Patients (n = 155)	Arm 2: MI for Patients and Caregivers (n = 177)	Arm 3: Standard of Care (n = 178)	Arm 2 vs Arm 1		Arm 2 vs Arm 3	
		Mean (SD)	Mean (SD)	Mean (SD)	Difference (95%CI)	*P*	Difference (95%CI)	*P*
Δ in the Hospital Anxiety Scale^a^								
T1	319	−0.6 (3.0)	−0.7 (3.1)	−0.4 (3.6)	−0.09 (−0.92 to 0.74)	.8370	−0.31 (−1.18 to 0.57)	.4877
T2	280	−1.9 (4.6)	−1.5 (4.8)	−2.2 (4.8)	0.41 (−0.95 to 1.76)	.5533	0.67 (−0.69 to 2.03)	.3352
T3	248	−2.3 (4.8)	−1.9 (5.3)	−2.3 (5.1)	0.34 (−1.20 to 1.89)	.6597	0.34 (−1.24 to 1.93)	.6683
T4	232	−1.7 (4.8)	−1.8 (5.3)	−2.2 (5.1)	−0.15 (−1.76 to 1.46)	.8542	0.37 (−1.24 to 1.98)	.6523
Δ in the Hospital Depression Scale^a^								
T1	319	−0.5 (3.0)	−0.4 (3.0)	−0.1 (3.6)	0.18 (−0.64 to 0.99)	.6672	−0.21 (−1.10 to 0.67)	.6381
T2	280	−1.8 (4.9)	−1.1 (4.7)	−1.6 (5.0)	0.63 (−0.74 to 2.01)	.3628	0.42 (−0.95 to 1.80)	.5457
T3	248	−1.8 (5.0)	−1.2 (5.3)	−1.7 (5.6)	0.57 (−0.99 to 2.13)	.4736	0.49 (−1.16 to 2.14)	.5580
T4	232	−1.5 (5.1)	−1.4 (5.1)	−1.7 (5.4)	0.06 (−1.56 to 1.67)	.9446	0.29 (−1.36 to 1.93)	.7309
Δ in physical SF-12^a^								
T1	319	2.5 (6.9)	1.4 (6.7)	0.6 (7.5)	−1.09 (−2.94 to 0.77)	.2492	0.76 (−1.11 to 2.63)	.4234
T2	281	2.4 (7.1)	2.3 (8.0)	1.6 (7.3)	−0.04 (−2.23 to 2.16)	.9748	0.74 (−1.44 to 2.92)	.5022
T3	247	2.7 (6.4)	2.0 (7.5)	1.3 (7.4)	−0.66 (−2.81 to 1.49)	.5444	0.74 (−1.52 to 3.01)	.5195
T4	231	1.7 (7.7)	2.6 (8.5)	1.7 (6.3)	0.87 (−1.70 to 3.44)	.5035	0.94 (−1.37 to 3.24)	.4233
Δ in mental SF-12^a^								
T1	319	1.5 (7.2)	0.6 (7.7)	0.2 (9.1)	−0.85 (−2.89 to 1.20)	.4156	0.44 (−1.77 to 2.66)	.6955
T2	281	2.6 (9.4)	0.7 (7.8)	2.2 (8.2)	−1.88 (−4.33 to 0.57)	.1325	−1.52 (−3.79 to 0.76)	.1897
T3	247	3.6 (7.9)	1.7 (10.4)	2.7 (7.5)	−1.87 (−4.64 to 0.90)	.1834	−1.03 (−3.74 to 1.68)	.4662
T4	231	2.8 (8.0)	2.4 (10.0)	2.0 (8.4)	−0.38 (−3.20 to 2.45)	.7929	0.42 (−2.49 to 3.33)	.7750
Δ in global PSQI score^a^								
T1	312	−0.3 (2.8)	−0.5 (2.6)	0.4 (2.6)	−0.18 (−0.93 to 0.57)	.6354	−0.93 (−1.62 to −0.24)	.0086
T2	271	−0.7 (2.7)	−0.4 (3.0)	−0.6 (2.5)	0.33 (−0.50 to 1.16)	.4366	0.21 (−0.58 to 1.01)	.5977
T3	245	−0.7 (2.8)	−0.4 (2.7)	−0.6 (2.7)	0.25 (−0.59 to 1.08)	.5625	0.20 (−0.63 to 1.03)	.6402
T4	232	−0.8 (2.3)	−0.1 (2.8)	−0.7 (2.1)	0.69 (−0.12 to 1.51)	.0957	0.61 (−0.14 to 1.37)	.1108

T1, T2, T3, and T4 correspond to 3, 6, 9, and 12 months from enrollment, respectively.

Abbreviations: CI, confidence interval; MI, motivational interviewing; PSQI, Pittsburgh Sleep Quality Index; SF-12, 12-item Short Form Health Survey.

^a^Δ scores. The columns for each arm report the delta (Δ) of each score computed by subtracting the corresponding score at baseline from the corresponding score at each follow-up time (T1, T2, T3, T4).

**TABLE 3:** Longitudinal Linear Mixed-Model Results on Caregivers' Hospital Anxiety and Depression Scale, Physical and Mental 12-Item Short Form Health Survey, and Global Pittsburgh Sleep Quality Index Scores

	β	95% CI	*P*
Hospital Anxiety Scale			
Difference for each visit (arm 2: MI for patients and caregivers)	−0.45	−0.67 to −0.24	<.0001
MI for patients and caregivers (arm 2) vs MI only for patients (arm 1) at baseline	−0.44	−1.28 to 0.41	.3100
MI for patients and caregivers (arm 2) vs standard of care (arm 3) at baseline	−0.32	−1.13 to 0.48	.4269
MI for patients and caregivers (arm 2) vs MI only for patients (arm 1) at follow-up	0.04	−0.28 to 0.36	.8160
MI for patients and caregivers (arm 2) vs standard of care (arm 3) at follow-up	0.09	−0.22 to 0.41	.5695
Caregiver living with the patient vs not living with the patient	0.63	−0.05 to 1.30	.0692
Hospital Depression Scale			
Difference for each visit (arm 2: MI for patients and caregivers)	−0.39	−0.60 to −0.18	.0003
MI for patients and caregivers (arm 2) vs MI only for patients (arm 1) at baseline	0.16	−0.64 to 0.97	.6894
MI for patients and caregivers (arm 2) vs standard of care (arm 3) at baseline	−0.33	−1.10 to 0.43	.3942
MI for patients and caregivers (arm 2) vs MI only for patients (arm 1) at follow-up	0.05	−0.27 to 0.36	.7659
MI for patients and caregivers (arm 2) vs standard of care (arm 3) at follow-up	−0.02	−0.33 to 0.29	.9133
Caregiver living with the patient vs not living with the patient	0.81	0.17–1.45	.0133
Physical SF-12			
Difference for each visit (arm 2: MI for patients and caregivers)	0.46	0.12–0.80	.0076
MI for patients and caregivers (arm 2) vs MI only for patients (arm 1) at baseline	−0.13	−1.75 to 1.48	.8743
MI for patients and caregivers (arm 2) vs standard of care (arm 3) at baseline	0.94	−0.60 to 2.47	.2304
MI for patients and caregivers (arm 2) vs MI only for patients (arm 1) at follow-up	0.00	−0.50 to 0.51	.9915
MI for patients and caregivers (arm 2) vs standard of care (arm 3) at follow-up	0.23	−0.27 to 0.72	.3683
Caregiver living with the patient vs not living with the patient	−2.75	−4.04 to −1.46	<.0001
Mental SF-12			
Difference for each visit (arm 2: MI for patients and caregivers)	0.64	0.23–1.04	.0021
MI for patients and caregivers (arm 2) vs MI only for patients (arm 1) at baseline	1.13	−0.65 to 2.92	.2142
MI for patients and caregivers (arm 2) vs standard of care (arm 3) at baseline	1.68	−0.02 to 3.37	.0527
MI for patients and caregivers (arm 2) vs MI only for patients (arm 1) at follow-up	−0.18	−0.79 to 0.42	.5518
MI for patients and caregivers (arm 2) vs standard of care (arm 3) at follow-up	0.19	−0.41 to 0.78	.5426
Caregiver living with the patient vs not living with the patient	−0.64	−2.06 to 0.77	.3733
Global PSQI score			
Difference for each visit (arm 2: MI for patients and caregivers)	−0.10	−0.22 to 0.02	.1146
MI for patients and caregivers (arm 2) vs MI only for patients (arm 1) at baseline	0.16	−0.53 to 0.84	.6527
MI for patients and caregivers (arm 2) vs standard of care (arm 3) at baseline	0.10	−0.55 to 0.76	.7569
MI for patients and caregivers (arm 2) vs MI only for patients (arm 1) at follow-up	0.10	−0.07 to 0.28	.2519
MI for patients and caregivers (arm 2) vs standard of care (arm 3) at follow-up	0.08	−0.10 to 0.25	.3879
Caregiver living with the patient vs not living with the patient	0.15	−0.40 to 0.69	.5940

Arm 2 was set as the reference in the model, but coefficient estimates are expressed with reference to arms 1 and 3 to be consistent with Table 2.

Abbreviations: CI, confidence interval; MI, motivational interviewing; PSQI, Pittsburgh Sleep Quality Index; SF-12, 12-item Short Form Health Survey.

### Caregiver Physical and Mental Quality of Life at Follow-ups

Table 2 shows the changes (Δ) in caregivers' physical and mental QoL at each follow-up in each study arm. Over the year of the study, these 2 variables increased slightly over time, but without significant differences among the 3 arms. Model-based trends showed that, over the year of the study, caregiver physical and mental QoL scores increased nearly one-half point for each visit (increase of PCS in arm 2: 0.46 [95% CI, 0.12–0.80], *P* = .0076; increase of MCS in arm 2: 0.64 [95% CI, 0.23–1.04], *P* = .002). The increment was not different in the 3 arms, with the 95% CIs of the coefficients estimating the difference between arm 2 as compared with arms 1 and 3 during follow-up lying entirely over −0.8. This means that the maximum estimated difference in favor of arms 1 and 3 was −0.8 for each follow-up visit (Table 3). Over the year of the study, caregivers living with the patient reported a lower PCS (−2.75; 95% CI, −4.04 to −1.46; *P* < .0001; Table 3), compared with caregivers not living with the patient, but not MCS (−0.64; 95% CI, −2.06 to 0.77; *P* = .3733; Table 3).

### Caregiver Sleep Quality at Follow-up

Changes (Δ) in caregiver sleep quality are shown in Table 2. Over the follow-up period, caregiver sleep disturbances decreased in all 3 arms, but more strongly in arms 1 and 3 than in arm 2. From baseline to T1, caregiver mean sleep disturbances decreased more in arm 2 compared with arm 3 (difference, −0.93; 95% CI, −1.62 to −0.24; *P* = .008), but the difference shrank during follow-up. When the 3 arms were analyzed in the longitudinal model, no differences in PSQI were identified among the 3 arms over time. Over the year of the study, caregivers living with the patient had higher sleep disturbances, compared with caregivers not living with the patient, although the difference was not statistically significant (0.15; 95% CI, −0.40 to 0.69; *P* = .59).

## Discussion

The aim of this study was to evaluate whether an MI intervention aimed at improving CC to HF self-care affected caregiver anxiety, depression, QoL, and sleep. We found that our intervention, in which caregivers were guided to improve their support toward patient self-care, did not increase caregivers' levels of anxiety and depression and did not decrease their QoL and sleep. These findings are important for several reasons. First, literature reports that taking care of a person with HF is a burdensome experience for caregivers^9^ and relying on caregivers for patient care could worsen caregivers' health status. However, in our case, similar to the Wingham et al^14^ study, encouraging caregivers to improve their contribution to patient self-care did not worsen caregiver outcomes. Second, because better CC to self-care is known to be associated with better patient outcomes,^9,31^ researchers should try to improve CC to patient self-care. In that sense, our findings suggest that relying on caregivers to support patients' self-care does not worsen caregivers' own condition. Therefore, researchers may safely ask caregivers to contribute to patient self-care. If these findings were confirmed by other studies, they could help healthcare providers to tailor future interventions in HF care, with an emphasis on caregiver involvement in patient self-care.

Consistent with those from the REACH-HF trial,^14^ our results previously showed that the intervention improved caregiver self-efficacy, but not CC to HF self-care.^32^ In this study, we also added that the intervention did not worsen caregiver anxiety, depression, QoL, and sleep quality. These findings may mean that caregivers' anxiety, depression, QoL, and sleep did not significantly change because MI did not significantly improve CC to self-care. Second, it could be that the significant improvement of caregiver self-efficacy prevented these variables (caregiver anxiety, depression, QoL, sleep) from worsening. Indeed, previous theories^9^ and studies^33^ have already underlined the key role of caregiver self-efficacy by showing it as a mediator between predictors of CC to self-care (eg, personal characteristics of caregivers) and CC to self-care itself. Third, it may be that delivering interventions to caregivers to improve their contribution to patient self-care does not worsen caregivers' anxiety, depression, QoL, or sleep.

Little evidence exists on the impact of interventions to improve CC to self-care on caregivers' own anxiety, depression, QoL, and sleep in HF. Indeed, 1 trial^34^ examined the effect of a 3-month multidisciplinary supportive program for caregivers of patients with HF on caregivers' own QoL and depression. That study conducted in China found that caregivers in the intervention group reported higher mental QoL and lower depression. These results suggest that the intervention not only did not worsen caregivers' QoL and depression, as we found, but also improved QoL and depression. However, CC to self-care was not measured in that study, making it impossible to know whether it changed. Future research is needed to further investigate the relationship between CC to self-care and caregivers' outcomes.

Over the year of the study, we found that caregivers living with the patients reported significantly lower physical QoL and higher depression scores than those not living with the patients. Our interpretation is that living with patients with HF is a burdensome experience for caregivers, who have to undertake several tasks to support their loved ones, resulting in their physical and emotional perceptions to be negatively affected.

### Limitations

This study has limitations. At the 12-month follow-up, we observed a 55.8% attrition rate in caregivers due to refusal to continue in the study or to patient death. Nevertheless, to the best of our knowledge, this is the trial in which the biggest sample of HF caregivers received an MI intervention. Indeed, at 12 months, our final sample included 235 caregivers, among which 177 received the intervention. We adjusted for the dropout rate using a linear mixed model that includes all the subjects randomized in an intention-to-treat principle. In addition, our MI intervention did not reach the highest possible quality in terms of its technical and relational components, despite the initial training provided to the interventionist nurses. This may suggest that the interventionists should be offered a stronger initial training or the chance to timely report and address potential difficulties in the delivery of the MI. Perhaps if the MI intervention was more robust, the caregivers receiving the intervention could even have improved their anxiety, depression, QoL, and sleep. However, we assessed MI quality and, to the best of our knowledge, no other study has performed such assessment.

## Conclusion

The results of this study show that delivering an MI intervention addressing CC to self-care does not increase caregiver anxiety and depression and does not decrease caregiver QoL and sleep. Therefore, this might suggest that such an intervention may be safely delivered to HF caregivers without worsening their condition. However, further studies are needed to confirm our results.

### Relevance to Clinical Practice

Because an intervention aimed at improving CC to self-care does not seem to have a negative impact on HF caregiver anxiety, depression, QOL, and sleep, informal caregivers may be encouraged to help patients with HF to perform better self-care. Available evidence shows that higher CC to HF self-care is associated with better levels of medication adherence, exercise, diet, and flu vaccination,^10^ and lower mortality rates and rehospitalizations.^11^ Consequently, educating the caregiver to support patient self-care could be a less expensive and effective approach to reducing the poor outcomes associated with HF.

**What’s New and Important**

- Caregiver contribution to self-care in HF does not seem to increase caregiver anxiety and depression and also does not seem to decrease their QoL and sleep.
- Clinicians might safely ask caregivers to take an active role in contributing to patient self-care, while still monitoring caregivers' conditions to prevent detrimental effects on caregivers' QoL.
